# The contribution of intermittent handheld electrocardiogram and continuous electrocardiogram monitoring with an implantable loop recorder to detect incident and recurrent atrial fibrillation during 1 year after coronary artery bypass graft surgery: A prospective cohort study

**DOI:** 10.1016/j.hroo.2021.05.001

**Published:** 2021-05-11

**Authors:** Emma Sandgren, Anders Wickbom, Torbjörn Kalm, Anders Ahlsson, Nils Edvardsson, Johan Engdahl

**Affiliations:** ∗ Department of Clinical Sciences, Karolinska Institutet, Danderyd University Hospital, Stockholm, Sweden; † Department of Medicine, Halland Hospital Varberg, Varberg, Sweden; ‡ Department of Cardiothoracic and Vascular Surgery, Örebro University Hospital, Örebro, Sweden; § Department of Cardiothoracic Surgery, Karolinska University Hospital, Stockholm, Sweden; ‖ Sahlgrenska Academy at Sahlgrenska University Hospital, Gothenburg, Sweden

**Keywords:** Atrial fibrillation, Coronary artery bypass graft surgery, ECG monitoring, Implantable loop recorder, Handheld ECG

## Abstract

**Background:**

Atrial fibrillation (AF) is common after coronary artery bypass graft (CABG) surgery.

**Objective:**

To evaluate the incidence and recurrence rate of AF during 1 year after CABG surgery. We also aimed at calculating the AF burden and compare long-term intermittent vs continuous electrocardiogram (ECG) monitoring.

**Methods:**

Forty patients scheduled for CABG surgery were equipped with an implantable loop recorder (ILR). After discharge, they carried out handheld ECG 3 times daily during the first 30 postoperative days and during 2 weeks at 3 and 12 months. During hospital stay they were monitored with telemetry.

**Results:**

Altogether 27 of 40 (68%) patients were diagnosed with AF, 24 during the first month (21 in-hospital and 3 after discharge) and 3 during months 2–12. Three patients progressed into persistent AF. In addition, 17 patients had AF recurrence, 9 of them after the first 30 days. In patients with paroxysmal AF, the AF burden was low, 0.1% (interquartile range [IQR] 0.02%–0.3%). Patients with AF had higher CHA_2_DS_2_-VASc scores than non-AF patients: median 4 (IQR 3–4) and 3 (IQR 2–3.5), respectively, *P* = .006. The handheld ECG identified 45% (9/20) of the patients with AF episodes identified with continuous ECG monitoring with the ILR after discharge from hospital, *P* = .001.

**Conclusions:**

Patients with AF during the postoperative hospitalization showed a high likelihood of recurrent AF, usually within 30 days. Continuous ECG monitoring with an ILR was superior to the handheld ECG for detecting patients with AF. The AF burden was low.

## Graphical abstract

**Figure:**
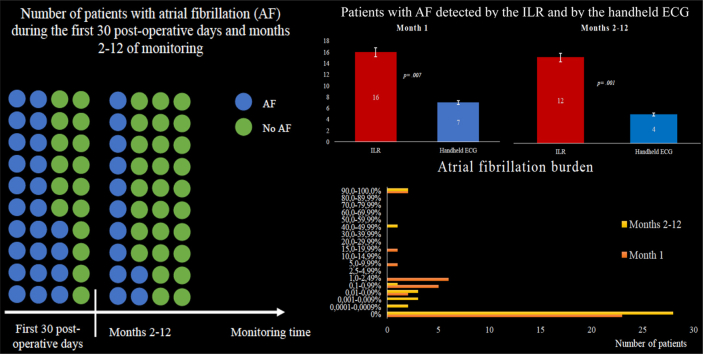

**Key Findings**

▪ Episodes of atrial fibrillation (AF) were common, especially during the first 30 postoperative days.
▪ The AF burden was low.
▪ Patients with any AF had higher CHA_2_DS_2_-VASc score than non-AF patients.
▪ Continuous electrocardiogram (ECG) monitoring with an implantable loop recorder was superior to noninvasive handheld ECG monitoring in detecting patients with AF.

## Introduction

Coronary artery bypass graft (CABG) surgery is commonly associated with episodes of atrial fibrillation (AF), both during the immediate postoperative period and later on.^1^, ^2^, ^3^, ^4^ The proportion of patients with AF during inpatient care after CABG surgery is in the range of 20%–40%.^1^^,^^2^^,^^5^ In the long term, these patients have an increased risk for AF, ischemic stroke, and cardiovascular mortality compared to patients who remain in sinus rhythm after CABG surgery.^3^^,^^5^, ^6^, ^7^, ^8^, ^9^, ^10^

Data from an observational study suggested that anticoagulation treatment may improve survival in patients with incident AF after CABG surgery,^11^ but there is no evidence from randomized trials that these patients have a net benefit from anticoagulation treatment. In guidelines from the European Society of Cardiology (ESC) and the American College of Cardiology/American Heart Association/Heart Rhythm Society (ACC/AHA/HRS) there are class IIb and class IIa recommendations, respectively, to consider long-term anticoagulation treatment in these patients in the presence of other stroke risk factors (ie, hypertension, age, diabetes, vascular disease, or prior ischemic stroke).^12^, ^13^, ^14^

Short transient and asymptomatic episodes of AF are difficult to detect with conventional electrocardiogram (ECG) recording methods. The detection rate increases with increased monitoring frequency, dispersion, and duration.^15^ Long-term intermittent screening with handheld ECG is noninvasive, available, and possible to perform several times daily and has high sensitivity and specificity for AF detection.^16^ However, the method is dependent on patient compliance and ability to follow instructions. An implantable loop recorder (ILR), on the other hand, is not dependent on patient capacity (but invasive), has the capacity of long-term continuous ECG monitoring, and has higher sensitivity for AF detection than noninvasive long-term ECG modalities.^15^

The primary aim of this study was to test the hypothesis that patients with incident AF during the postoperative hospitalization period often relapse in AF within a year, with little chance of detection. Secondary aims were to calculate the AF burden among the patients with paroxysmal AF episodes and compare the efficacy of long-term intermittent (handheld ECG) vs continuous (ILR) ECG monitoring in detecting patients with AF.

## Patients and methods

This was a substudy of the prospective AFAF study (Atrial Fibrillation AFter CABG and percutaneous coronary intervention; NCT04307225). The AFAF study investigates the incidence of AF in 250 patients using noninvasive handheld ECG recordings (Zenicor-EKG®; Zenicor Medical Systems AB, Stockholm, Sweden) 3 times daily during the first month following the revascularization procedure and thereafter for 2 weeks at 3, 12, and 24 months after surgery, in addition to routine care. The patients were instructed to perform the ECG recordings at the same times every day, as well as in case of symptoms. Each ECG recording was 30 seconds. All recordings were stored in a central database available only for investigators.

Patients who were scheduled for CABG surgery and were eligible for participation in the main study were also asked to participate in the present study, which in addition to the handheld ECG included continuous ECG monitoring with an ILR (Medtronic Reveal LINQ; Medtronic, Minneapolis, MN). A total of 105 patients were asked to participate, 40 of whom agreed to participate. The ILRs were implanted subcutaneously in the parasternal region during the CABG surgery. Before discharge data collection was activated and remote monitoring instituted.

Exclusion criteria were a history of AF, pacemaker treatment or other non–sinus rhythm, bleeding disorder where treatment with oral anticoagulation was contraindicated, cognitive impairment or communication problems leading to difficulties in taking instructions and filling in the written informed consent form, malignancy or other disease with life expectancy <1 year, and ongoing anticoagulation treatment.

Information about patient demographics, comorbidity, classification of coronary artery disease (ie, stable angina, unstable angina, or non-ST-elevation myocardial infarction), disease degree (ie, number of vessels occluded), echocardiographic measurements (ie, left atrial area and left ventricular ejection fraction), and medications were retrieved from patient interviews, medical records, and digital report forms completed by phone at baseline and after 1, 3, and 12 months of follow-up. Information about the occurrence of AF during the hospital stay were retrieved from medical records, and to diagnose AF at least a 30-second telemetry recording or a 12-lead ECG was required, in accordance with ESC 2020 AF guidelines.^12^

The ILR was programmed to detect tachyarrhythmia >176 beats/min during at least 16 beats, pause >3 seconds, and bradyarrhythmia <30 beats/min during at least 4 beats. Algorithms for AF and atrial tachycardia were activated. The AF detection algorithm required at least 2 minutes of continuous AF to be captured. In addition to the predefined automatically captured arrhythmias, recordings could be performed by manual activation of the device by the patient or a bystander, eg, during symptoms. ILR data were checked weekly by investigators via remote monitoring. The patients were followed for 12 months.

### Outcome measures

The primary endpoint of the study was the proportion of patients that were diagnosed with incident or recurring AF during the 12-month follow-up. Secondary endpoints were the proportion of patients who developed persistent AF and the AF burden, calculated as the total time in AF during the 12-month follow-up. AF burden was determined based on all adjudicated >2-minute recordings in AF. ECG strips captured as AF were manually adjudicated (ES and JE) in order to exclude false-positive strips from analysis. On the handheld ECG, 30 seconds of AF was sufficient for an AF diagnosis.

Any other significant arrhythmias were recorded, eg, atrial flutter or atrial tachycardia according to their algorithms, or other arrhythmias when the device was manually activated owing to symptoms.

### Ethics

The study was approved by the Regional Ethical Review Board of Uppsala (Dnr 2015/413) and conformed to the ethical principles for medical research of the World Medical Association adopted in the Declaration of Helsinki. Patient consent was obtained through written informed consent form.

### Statistical analysis

Primary and secondary outcome variables are categorical and were reported as frequencies and percentages. Univariate analysis was conducted using χ^2^ test or Fisher exact test. Continuous variables were reported as either means and standard deviations or 95% confidence interval (CI) or medians and interquartile range (IQR). For statistical comparison a Student *t* test or the nonparametric Mann-Whitney *U* test were used. Logistic regression was performed to calculate adjusted odds ratios for variables predicting the occurrence of AF during the 12-month monitoring period after CABG surgery, including the variables in Table 1. The Enter method was used and variables with a probability value less than 0.1 were included in the final models. Collinearity diagnostics were performed with the variance inflation factor with a cut-off less than 2. Cox and Snell R^2^ and the Nagelkerke R^2^ values provided an indication of the amount of variation in the dependent variable explained by the model. Two-tailed tests were applied. *P* < .05 was regarded as significant. Data processing and analyses were carried out using Microsoft Excel and IBM SPSS version 27.0 (SPSS, Chicago, IL).

**Table 1:** Baseline characteristics in patients with and without atrial fibrillation during the 12-month monitoring after coronary artery bypass graft surgery

	No AF	AF	*P* value
Total number	13	27	
Age	63.6 ± 9.0	70.4 ± 6.9	.012∗
Women	0 (0%)	1 (3.7%)	1.0
Body mass index	29.3 ± 4.8	30.1 ± 5.3	.650
CHA_2_DS_2_-VASc score	3 (IQR 2–3.5)	4 (IQR 3–4)	.006∗
Smoking status			.200
Nonsmoker	8 (62%)	11 (40.7%)	
Former smoker (>1 month)	5 (38%)	11 (40.7%)	
Smoker	0 (0%)	5 (18.5%)	
Alcohol consumption†			.320
None	1 (8%)	3 (11.5%)	
<8 units/week	10 (84%)	23 (88.5%)	
>8 units/week	1 (8%)	0 (0%)	
Congestive heart failure	1 (7.7%)	6 (22.2%)	.390
Previous myocardial infarction	3 (23%)	16 (59.3%)	.046∗
COPD	0 (0%)	0 (0%)	1.0
Obstructive sleep apnea	1 (7.7%)	1 (3.7%)	1.0
Hypertension	7 (54%)	22 (81.5%)	.130
Diabetes	5 (38%)	10 (37%)	1.0
Previous stroke‡	0 (0%)	4 (16%)	.240
Peripheral vascular disease	0 (0%)	3 (11.1%)	.540
Disease definition			.690
Unstable angina	4 (31%)	5 (18.5%)	
Stable angina	7 (54%)	17 (63%)	
Non-STEMI	2 (15%)	5 (18.5%)	
Extent of disease (number of vessels)			.540
1	0 (0%)	0 (0%)	
2	0 (0%)	3 (11.1%)	
3	13 (100%)	24 (88.9%)	
Echocardiography			
Left ventricular ejection fraction	53.1 ± 8.5	50.9 ± 13.2	.600
Left atrium area§	20.7 ± 3.0	23.7 ± 5.0	.063
AF during hospitalization	6.5 ± 2.7	6.9 ± 3.3	.770

AF = atrial fibrillation; COPD = chronic obstructive pulmonary disease; IQR = interquartile range; STEMI = ST-elevation myocardial infarction.

Reported values are n (%), mean ± standard deviation, and median (IQR). Statistical tests used: Student *t* test, nonparametric Mann-Whitney *U* test, χ^2^ test, and Fisher exact test.

∗ *P* values indicate statistical significant.

† Data are missing for 1 patient with no AF and 1 patient with AF.

‡ Data are missing for 2 patients with AF.

§ Data are missing for 1 patient with no AF and 2 patients with AF.

## Results

### Study population

Forty patients were included, 39 men and 1 woman, with a mean age of 68 ± 8 years. Baseline characteristics for the patients with and without any AF during the 12-month monitoring period are presented in Table 1. Patients with incident AF had a higher CHA_2_DS_2_-VASc score than non-AF patients, median 4 (IQR 3–4) and median 3 (IQR 2–3.5), respectively, *P* = .006. Seven patients had had a non-ST-elevation myocardial infarction, 9 had unstable angina pectoris, and 24 had stable angina pectoris. CABG surgery was performed owing to 3-vessel disease (n = 37) and 2-vessel disease (n = 3). Peroperative complications occurred in 2 patients (pericardial effusion and cardiac ischemia) and were treated with no sequelae. All but 2 patients were alive and were followed until the end of the 12-month follow-up. These 2 patients were 62 and 69 years old and died 3 and 4 months after surgery, owing to, respectively, myocardial infarction and an unknown cause of death, since death occurred abroad.

### Incidence of AF

**Figure 1:**
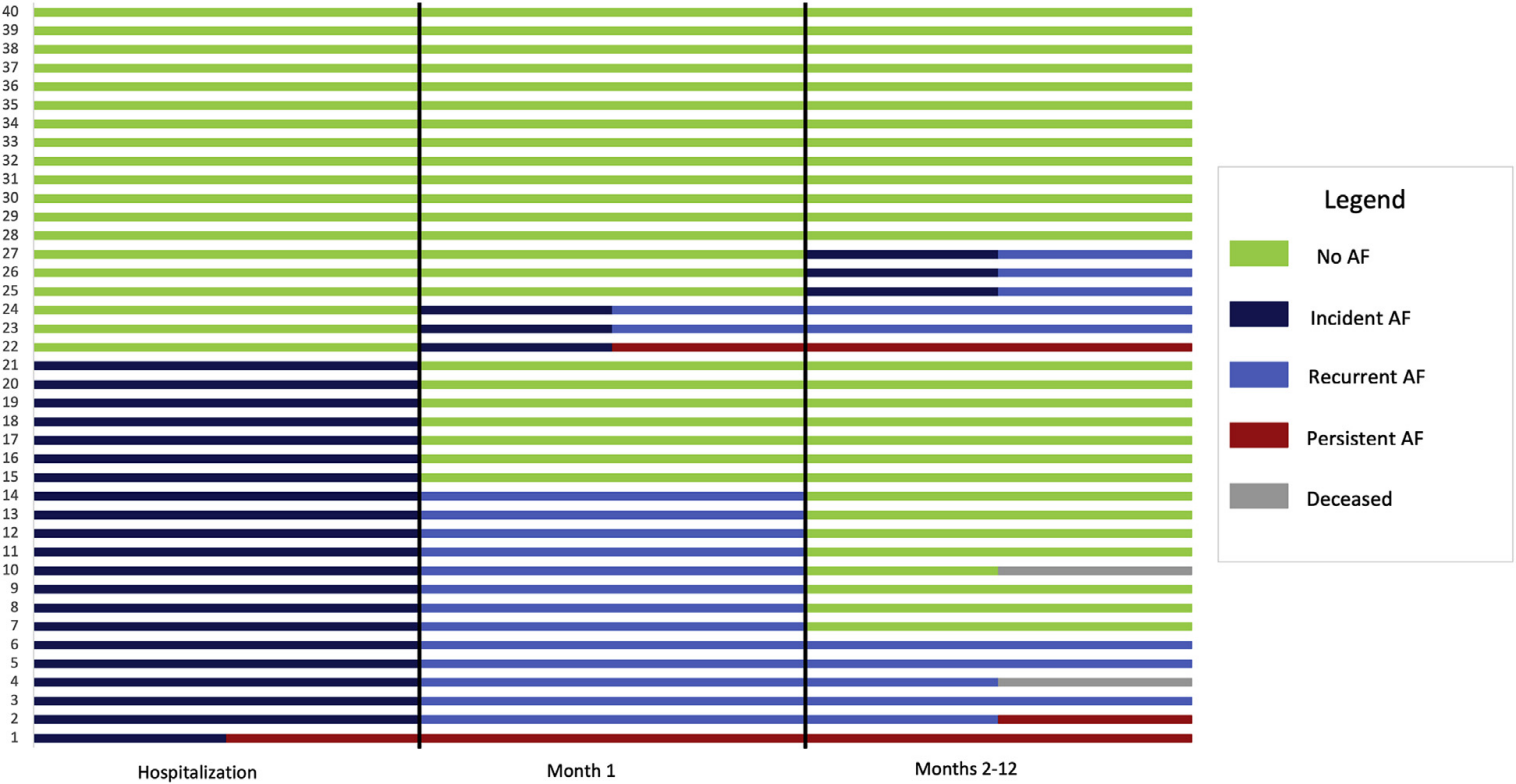
Incident and recurrence of atrial fibrillation (AF) captured by any method. In total 27 of 40 patients had incident AF. Three progressed into persistent AF and a further 17 had 1 or more recurrences of AF during follow-up.

The incidence and recurrence of AF is shown in Figure 1. During the postoperative in-hospital period incident AF occurred in 21 patients and all patients were detected with continuous ECG telemetry. The ILR was activated at discharge in all but 1 patient. After discharge, but within the first 30 postoperative days, another 3 patients had incident AF; hence 24 of 40 patients (60%) experienced incident AF during the first 30 postoperative days. During the remainder of the 12-month monitoring period, 3 of the remaining 16 patients had incident AF (after 2 months in 2 patients and after 9 months in 1 patient), resulting in a total of 27 of 40 patients (68%) with incident AF at any time. Of those with AF during the index hospital stay, 20 of 21 were in sinus rhythm at discharge.

### Recurrence of AF

The incidence and recurrence of AF is shown in Figure 1. Of the 24 patients with incident AF during the first 30 postoperative days, 2 patients developed persistent AF (as first episode during hospitalization and as first episode after 9 days, respectively) and 7 experienced recurrence of AF during months 2–12 of monitoring and 1 of them progressed to persistent AF. All 3 patients with incident AF after the first 30 postoperative days had recurring episodes of AF.

### Pharmacological treatment

Thirty-three of 40 patients were on beta-blocker therapy prior to CABG surgery, including 18 of the 21 with AF during the index hospital stay. Seventeen of the 21 patients with incident AF during hospitalization received intravenous amiodarone, but only 3 patients were prescribed antiarrhythmic treatment (1 amiodarone, 1 amiodarone plus direct current cardioversion, and 1 dronedarone) during the first month owing to episodes of AF. No new antiarrhythmic treatment was instituted during months 2–12. All patients diagnosed with AF were prescribed anticoagulation treatment.

### Baseline predictors of any AF during the 12-month monitoring after CABG surgery

Logistic regression was used to identify variables predicting the incidence of AF. The final model contained 4 independent variables—age, hypertension, previous myocardial infarction, and left atrium area—and was statistically significant: *x*^2^ (4, N = 37) = 16.8, *P* = .002. The model explained 37%–51% of the variance and correctly classified 78.4% of cases. After adjustment, only age made a unique statistically significant contribution to the model (Table 2).

**Table 2:** Logistic regression for prediction of the likelihood of any atrial fibrillation during the 12-month monitoring period after coronary artery bypass graft surgery

	Unadjusted odds ratio (95% CI)	Adjusted odds ratio (95% CI)	*P* value
Age	1.1 (1.02–1.24)	1.2 (1.01–1.36)	.043
Hypertension	3.8 (0.88–16.24)	8.4 (0.95–74.09)	.055
Previous MI	4.8 (1.08–21.76)	4.4 (0.57–33.68)	.155
Left atrium area	1.2 (0.98–1.46)	1.2 (0.91–1.47)	.243

CI = confidence interval; MI = myocardial infarction.

Statistical test used: binary logistic regression.

### Other ILR-detected arrhythmias

In 1 patient 1 episode with asymptomatic nonsustained monomorphic ventricular tachycardia of 23 beats at a heart rate of 200 beats/min was recorded, while 7 patients had asymptomatic bradyarrhythmia (1 intermittent atrioventricular block III and 6 sinus arrest or sinus bradycardia). All episodes were detected by the predefined heart rate algorithms and none of these were captured with the handheld ECG.

### Diagnostic yield of an ILR compared to a noninvasive handheld ECG

**Figure 2:**
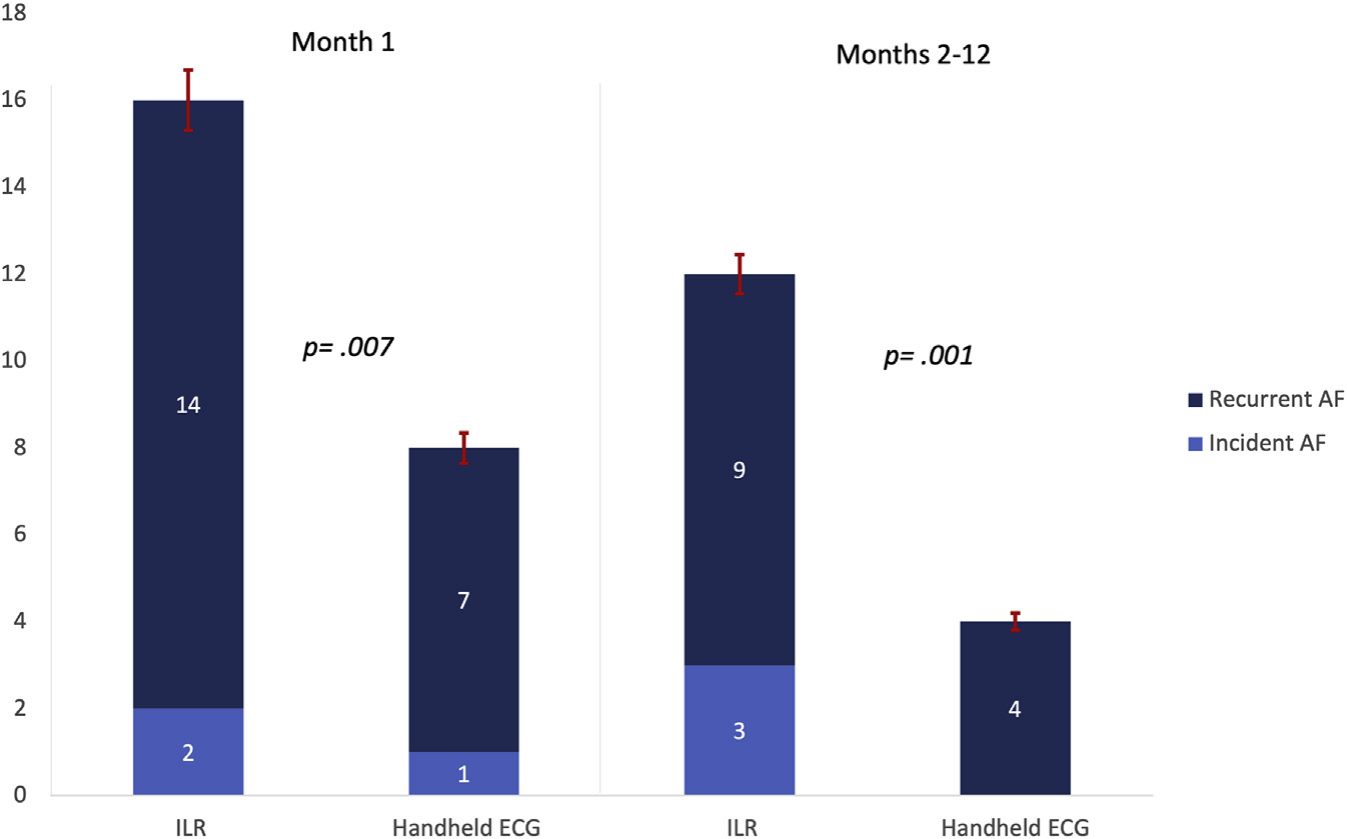
Number of patients with incident and/or recurrent atrial fibrillation (AF) detected by the implantable loop recorder (ILR) and the handheld electrocardiogram (ECG), respectively, during month 1 and months 2–12 of monitoring. One patient with incident AF found by the handheld ECG during the first month was not detected by the ILR, since the ILR had not been activated at discharge. The detection rate was significantly higher for the ILR than the handheld ECG for month 1 and months 2–12, 94% (16/17) vs 47% (8/17), *P* = .007 and 100% (12/12) vs 33% (4/12), *P* = .001, respectively. The error bars show the 95% confidence interval for the detected proportion. Statistical test used: Fisher’s exact test.

After discharge, 20 of the 40 patients (50%) had episodes of AF during the 12-month monitoring identified by the ILR, while 9 (22.5%) were identified by the handheld ECG, *P* = .001. In the first week after discharge, 1 patient had 2 AF episodes captured with the handheld ECG only, since the ILR had not been activated at discharge as intended. The AF detection rate was significantly higher for the ILR compared to the handheld ECG during the first month and months 2–12 (Figure 2). Three patients developed persistent AF, which was captured with both the ILR and the handheld ECG.

In total, 104 episodes of AF occurred in the 20 patients during the 12-month postdischarge follow-up, 101 of them paroxysmal. One hundred and two (98%) episodes were detected by the ILR and 30 (29%) by the handheld ECG recordings, *P* < .0001.

In addition to the handheld ECG recording 3 times daily, 12 patients made in total 21 handheld ECG recordings owing to symptoms and none of them showed AF. On average the patients performed 2.83 handheld ECG recordings per monitoring day.

### AF burden calculated from ILR-detected AF episodes

**Figure 3:**
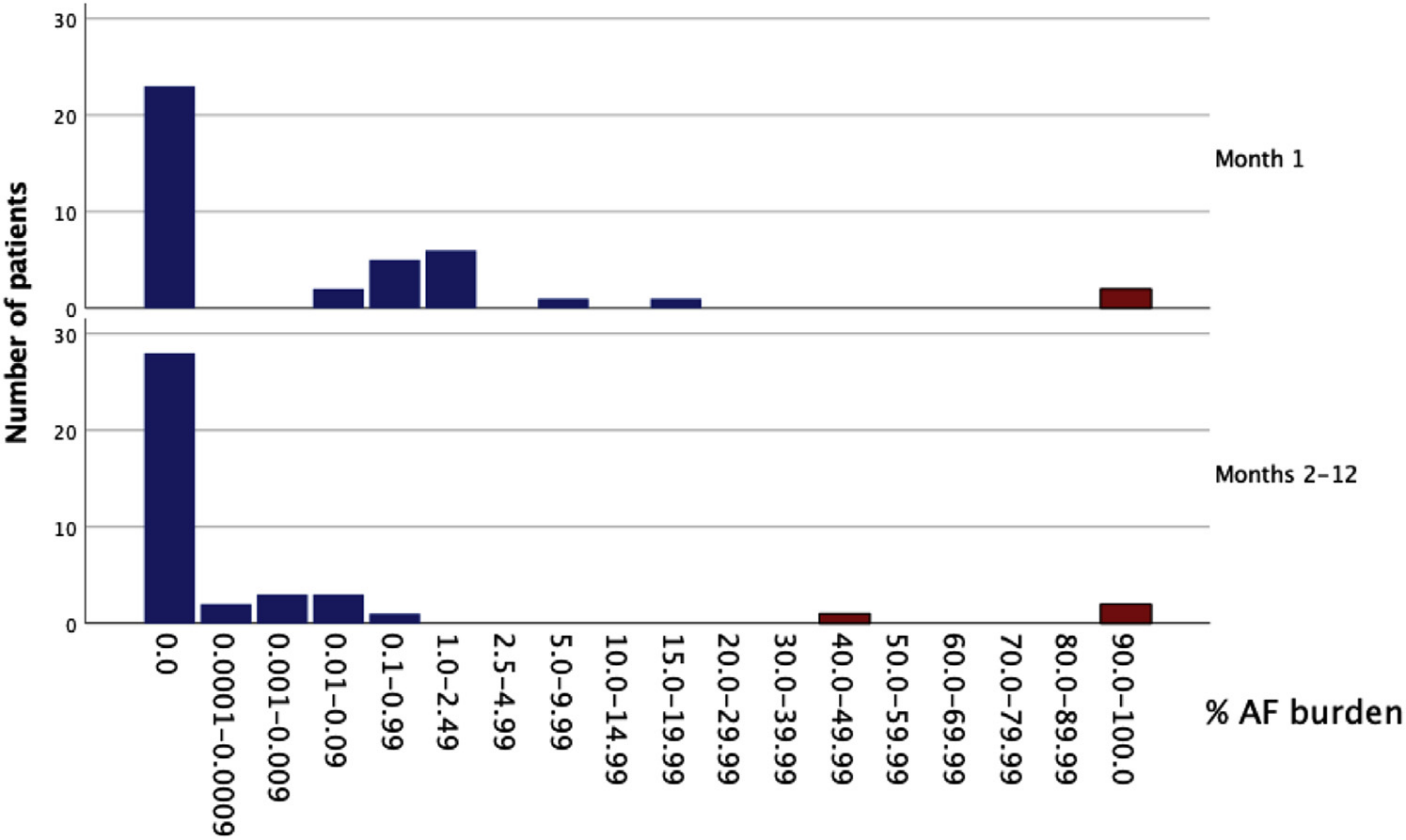
The atrial fibrillation (AF) burden was low except for the 3 patients (colored in red) who developed persistent AF, and it gradually decreased during the 12-month follow-up.

The AF burden was calculated from the duration of all adjudicated AF episodes lasting >2 minutes. The 3 patients with persistent AF were excluded from analysis. In the remaining patients with paroxysmal AF the median AF burden was 0.1% (IQR 0.02%–0.3%) with a minimum value of 0.003% and a maximum value of 1.5%, translating into 718 (IQR 136–1432) minutes (Figure 3). Among the 17 patients with paroxysmal AF after discharge, 14 had AF episodes with a duration of ≥6 minutes, while AF episodes ≥6 minutes accounted for 58 of the 101 paroxysmal AF episodes. More detailed data are found in supplementary materials (Supplemental Data File 1).

## Discussion

Both incident and recurrent episodes of AF were common, especially during the first 30 postoperative days. The AF burden in the cohort was mainly driven by persistent AF and the contribution from paroxysmal AF was small. Patients with any AF had higher CHA_2_DS_2_-VASc score than non-AF patients. Continuous ECG monitoring with an ILR was superior to noninvasive handheld ECG monitoring in detecting patients with AF during the 12-month monitoring after CABG surgery.

### Handheld intermittent ECG or continuous ECG monitoring with an ILR

Continuous ECG monitoring with an ILR identified that half of the patients in our study had incident or recurrent AF episodes during follow-up, while 45% of these patients were detected by a noninvasive handheld ECG. The ILR was also superior in finding individual AF episodes. The difference in AF detection rate between the 2 methods could possibly have been smaller if a more extended schedule (ie, daily) for handheld ECG recordings had been used.^17^, ^18^, ^19^ AF screening in general^20^ and handheld ECG in particular^21^ have been proven to be well tolerated, but most probably an extended handheld ECG recording schedule would have been at the expense of patient compliance.^20^ In addition, most AF episodes were of short duration and accordingly hard to capture with intermittent ECG monitoring methods. It is established that continuous ECG monitoring with an ILR captures significantly more paroxysmal episodes of AF than noninvasive modalities,^22^, ^23^, ^24^, ^25^ and routine clinical AF classification based on intermittent ECG monitoring poorly reflects AF temporal persistence.^26^ The ILR is capable of assessing the AF burden from the number and duration of individual AF episodes. In the ESC 2020 AF guidelines a structured characterization of AF is recommended, including the severity of AF burden, although the clinical benefit of this additional information has not yet been fully elucidated. Therefore, the guidelines emphasize the insufficient evidence between AF burden and AF-related outcome to guide treatment decisions.^12^

### Incidence of AF episodes

During the first 30 postoperative days two-thirds of the patients in our study had incident episodes of AF, which was a greater frequency than in earlier reports.^27^, ^28^, ^29^ The type and duration of monitoring have an impact on the yield. Bidar and colleagues^27^ used a 30-day event recorder and found an AF incidence of 49.3%, while Thorén and colleagues^28^ found an AF incidence of 42% over 30 days using a handheld ECG 3 times daily following in-hospital telemetry. Finally, Abdel-Salam and Nammas^29^ used a 15-day event recorder followed by 15 days of clinical follow-up and found an AF incidence of 10.4%.

### AF recurrence and progression

Among patients with AF during the in-hospital stay, AF recurrences were common during the first postoperative month. As many as two-thirds of patients in our study with AF during the hospitalization period continued to have episodes of AF in the first postoperative month. In a meta-analysis, including 6 studies, the incidence rate of AF recurrence with noninvasive monitoring in the first month after discharge was 28.3% (95% CI 23.0%–33.6%),^30^ which is comparable to our finding that 33% of patients with in-hospital AF had recurrent AF detected by a handheld ECG in the first month. In the MONITOR-AF study, 23 patients with AF during in-hospital stay were prospectively assigned to receive an ILR for detection of recurrent AF.^31^ During the first 3 months, 39% had recurrent AF, which was lower compared to our study. One reason for the difference could be the high proportion (96%) of patients with amiodarone at discharge in their study and that they defined AF as any episode lasting ≥6 minutes. Thus, recurrences of AF after discharge from hospital were common during the first month.

In our study population, the AF burden decreased after the first month, which could reflect the healing process where inflammation subsided. However, earlier studies have shown that incident AF after CABG surgery is associated with an increased long-term risk of developing AF^3^^,^^4^ or embolic stroke^6^^,^^10^ and even increased mortality.^3^^,^^32^ Thus, AF after CABG surgery might be a marker for patients with an existing or developing AF substrate; ie, atrial cardiomyopathy predisposed patients to develop AF during a provocation, such as CABG surgery, in the short term and spontaneously in the long term.

Interestingly, apart from the first postoperative month, there were equal proportions of patients with AF episodes among those with and without AF during the inpatient care, indicating that it is difficult to predict which patients are at increased risk for AF recurrence or AF incidence in the long term. These late-detected AF episodes may indicate that our study population constituted a high-risk group for developing AF owing to underlying cardiovascular comorbidity rather than related to prior CABG surgery.

During continuous monitoring, episodes of AF are common in patients with cardiovascular risk factors.^22^^,^^23^^,^^33^^,^^34^ In the REVEAL-AF study the incidence of AF was 27% after 12-month monitoring^34^ and in the LOOP study 35% had AF episodes lasting ≥6 minutes during a median of 40.2 months of monitoring.^23^ In the ASSERT-II study the detection rate of AF was 34.4% (95% CI 27.7%–42.3%) per year during 16.3 ± 3.8 months’ follow-up.^33^ In the latter study there was an association between age and incident AF.^33^ However, there were also associations with left atrium size and blood pressure, which we did not find, probably owing to type II error. It is important to find patients with AF in this population, as most of them will be considered eligible for anticoagulation treatment according to risk scores (ie, CHA_2_DS_2_-VASc).

There is still uncertainty about how these short episodes of AF affect stroke risk and there is no evidence from randomized trials that anticoagulation treatment is beneficial in patients with incident AF after CABG surgery in the long term. In addition, many patients undergoing CABG surgery have an indication of dual antiplatelet therapy, which also must be taken into account. Current guidelines recommend considering anticoagulation treatment in patients with incident AF and stroke risk factors, while weighing the risk of stoke vs the risk of bleeding. This treatment should have a minimum duration of 4 weeks after restoration of sinus rhythm. Prolonged monitoring can be beneficial in patients with high stroke risk according to the CHA_2_DS_2_-VASc score.^12^, ^13^, ^14^

### Clinical relevance of the AF burden calculated from ILR-detected AF episodes

In summary, 24 patients were identified with nonpersistent AF and could be evaluated for anticoagulation treatment. All of them had in common a low AF burden. A high prevalence of AF but a low AF burden in patients with stroke risk factors was also reported in the LOOP study, which reported a median AF burden of 0.13% (IQR: 0.03%–1.05%).^23^ Nevertheless, patients with newly detected AF as recorded by implantable devices commonly progress to higher AF burden,^35^ and a greater AF burden is associated with a higher risk of ischemic stroke.^36^ It seems reasonable that the combination of the AF burden and the risk factor score should be considered in the evaluation for anticoagulation treatment.^12^ It remains to be shown if information obtained from components of the AF burden (eg, the longest AF episode or number of episodes) would be of value in the decision-making on treatment.

### Limitations

In this prospective study all patients were recruited from 1 Swedish hospital, and the population may not be representative of those in other hospitals and regions. Forty of 105 patients who were asked chose to participate, and they may or may not be representative of the complete AFAF study population; therefore, we refrain from making any generalization.

Although both women and men were eligible for participation, the sex distribution was skewed; 2.5% were women in our study, compared to 10% in the total AFAF study population and 20% in the total Swedish CABG population (including patients with known AF before CABG surgery). The small sample size is a limitation and brings a degree of uncertainty as to its generalizability to the general population. Furthermore, the frequency of handheld ECG recordings (ie, 3 times daily during the first month and for 2 weeks at 3 and 12 months) used in the study had an impact on the diagnostic yield of the handheld ECG. Lastly, the patients were not monitored prior to surgery, and accordingly our definition of incident AF does not exclude the possibility that some patients might have had asymptomatic episodes of AF before inclusion.

## Conclusion

Half of the patients had incident AF during the postoperative hospitalization, and those patients showed a very high likelihood of having recurrent AF, usually within the first 30 postoperative days. The AF burden was low. Continuous ECG monitoring with an ILR was superior to the handheld ECG in detecting patients with episodes of AF after discharge, as the handheld ECG identified less than half of patients with AF.

### Clinical implications

Long-term monitoring with an ILR was superior to intermittent handheld ECG and demonstrated that while most AF episodes occurred within the first 30 postoperative days, incident AF occurred in 18% of the remaining patients thereafter and recurred in all of them within the observation period.

Postoperatively incident AF or recurrences seemed to cause few or no symptoms. With handheld ECG half of them were detected, but without continuous monitoring the other half would have remained undetected. At present, it remains to fully understand the risks of postoperative AF and the role of the AF burden, but if agreement were to be reached that more active treatment with anticoagulation is worthwhile (eg, in the patients with high risk scores), continuous long-term ECG monitoring would be the method of choice. Our results suggest that monitoring for at least 1 postoperative month provides a high yield, but that the rate of incident AF and recurrences beyond that time may not be negligible. Ideally, the results of this small study may be hypothesis-generating for a larger trial.

## Acknowledgments

The authors are grateful to Mariann Werling, RN, and Lars Forsell, RN, at the pacemaker unit and to research nurses Helen Hildingsson, RN, Karin Andersson, RN, and Anna Mattsson, RN; all at Örebro University Hospital. The proofreading was performed by Anchor English©.

## Funding Sources

Johan Engdahl was supported by the 10.13039/501100004348Stockholm County Council (clinical research appointment). Anders Wickbom was supported by the Örebro Regional Research Council and Nyckelfonden.

## Disclosures

ES, AW, TK, AA, and NE have nothing to declare. JE reports speaker and consultant fees from BioTel Europe, Boehringer Ingelheim, Bristol Myers Squibb, Medtronic, Merck Sharp & Dohme, 10.13039/100004319Pfizer, and 10.13039/100016545Roche Diagnostics.

## Authorship

All authors attest they meet the current ICMJE criteria for authorship.

## Patient Consent

Patient consent was obtained through written informed consent form.

## Ethics Statement

The study was approved by the Regional Ethical Review Board of Uppsala (Dnr 2015/413) and conformed to the ethical principles for medical research of the World Medical Association adopted in the Declaration of Helsinki.
